# Dermoscopy-Guided High-Frequency Ultrasound Imaging of Subcentimeter Cutaneous and Subcutaneous Neurofibromas in Patients with Neurofibromatosis Type 1

**DOI:** 10.3390/jcm15020475

**Published:** 2026-01-07

**Authors:** Krisztina Kerekes, Mehdi Boostani, Zseraldin Metyovinyi, Norbert Kiss, Márta Medvecz

**Affiliations:** 1 Department of Dermatology, Venereology and Dermatooncology, Faculty of Medicine, Semmelweis University, 1085 Budapest, Hungary; 2 Department of Dermatology, Roswell Park Comprehensive Cancer Center, Buffalo, NY 14203, USA

**Keywords:** neurofibromatosis, neurofibroma, high-frequency ultrasound, dermoscopy-guided high frequency ultrasound, dermoscopy-assisted high frequency ultrasound

## Abstract

**Background**: Neurofibromatosis type 1 (NF1) is an autosomal dominant disorder characterized by cutaneous and subcutaneous neurofibromas, which impact quality of life. Dermoscopy-guided high-frequency ultrasound (DG-HFUS) integrates dermoscopy with 33 MHz ultrasound, enabling precise lesion localization and reproducible measurements. Objective: To characterize neurofibromas in NF1 patients using DG-HFUS and identify imaging parameters for diagnosis, monitoring, and treatment planning. **Methods**: 14 genetically confirmed NF1 patients underwent DG-HFUS imaging (Dermus SkinScanner). 100 neurofibromas were assessed for size, location, shape, contours, surface, echogenicity, global echogenicity, and posterior acoustic features. **Results**: Lesions were dermal (79%) or subcutaneous (21%), round (28%), ovoid (63%), or spiked (9%). Mean vertical and lateral diameters were 5.37 ± 2.66 mm and 2.28 ± 1.39 mm. All were hypoechoic; 62% homogeneous, 38% heterogeneous. Margins were well-defined in 57% and poorly defined in 43%. Posterior enhancement occurred in 3% and shadowing in 10%. **Conclusions**: DG-HFUS provides a detailed, reproducible assessment of neurofibromas, supporting differential diagnosis, surgical planning, and longitudinal monitoring. The evaluated imaging parameters offer objective insights for optimizing NF1 management. Future developments, including 3D reconstruction and AI-assisted analysis, may further enhance its clinical utility.

## 1. Introduction

Neurofibromatosis type 1 (NF1) is an autosomal dominant genetic disorder affecting approximately 1 in 3000 individuals worldwide, irrespective of sex or ethnic background [1,2]. It is a neurocutaneous syndrome caused by pathogenic variants in the NF1 gene, which lead to the loss of functional neurofibromin. This protein normally acts as a negative regulator of RAS signaling. As a consequence, the RAS/RAF/MEK/ERK pathway becomes constitutively active, promoting cellular proliferation and playing a key role in tumor development [3]. In 50% of patients, family history is negative for NF1, and the disease results from a de novo NF1 mutation [4].

The clinical presentation of NF1 is highly diverse, with significant differences in symptoms and disease severity, even among individuals carrying the identical genetic alteration [5]. The literature indicates that characteristic clinical manifestations are present in approximately 50% of patients by the age of 1 year and in 97% by the age of 8 years [6]. The most frequent and earliest manifestations of the disease are cutaneous lesions. NF1 is characterized by multiple neurofibromas, café-au-lait macules (CALMs), axillary, inguinal, or diffuse freckling, and, less often, juvenile xanthogranuloma, nevus anemicus, or glomus tumour [7]. Among these cutaneous findings, neurofibromas are the hallmark lesions of NF1 [8]. These are benign nerve sheath tumors that occur in the vast majority of affected individuals and consist of Schwann cells, perineural cells, fibroblasts, mast cells, macrophages, neuronal axonal processes, and extracellular matrix components such as collagen [9]. They are classified into cutaneous, subcutaneous, and plexiform subtypes, each with distinct clinical and histopathological characteristics. The tumors may develop anywhere along peripheral nerves, their size ranging from a few millimeters to a few centimeters, and they may appear in large numbers, occasionally reaching hundreds or even thousands [1]. A characteristic feature known as the “buttonhole sign” refers to the invagination of the lesion when pressed [7]. Neurofibromas are often perceived by NF1 patients as their most significant burden due to visible disfigurement, which can lead to psychological distress and reduced quality of life (QOL) [8]. Additionally, pain and itching associated with neurofibromas may interfere with sleep, work, and social life, further diminishing QOL [8].

Beyond the characteristic dermatological manifestations, the clinical picture of NF1 commonly involves multiple organ systems, including neuropsychiatric, ophthalmological, musculoskeletal, cardiovascular, and endocrinological abnormalities, as well as an increased risk of various malignancies [4].

The diagnosis of NF1 is usually based on clinical findings; however, the use of molecular genetic testing is becoming more common to confirm the diagnosis. In May 2021, the diagnostic criteria for the disease were revised based on an international consensus recommendation, taking into account both the multidisciplinary clinical symptoms and the genetic background [10]. Molecular confirmation of the clinical diagnosis is increasingly important for distinguishing NF1 from related conditions, such as Legius syndrome, for exploring genotype–phenotype correlations and for identifying somatic mosaicism [11].

Molecular genetic testing in patients with NF1 primarily involves identifying a heterozygous pathogenic germline variant in the NF1 gene using DNA extracted from peripheral blood lymphocytes. CNV analysis is also recommended [12]. In mosaic or segmental forms of NF1, genetic analysis of tissue obtained from the affected area is required to establish the diagnosis [11].

Currently, there is no definitive cure for NF1, but with an accurate diagnosis, symptoms can be managed and patients can be regularly monitored in case more severe manifestations develop. Available treatment options for cutaneous and subcutaneous neurofibromas include electrodessication, photocoagulation, RF ablation, CO_2_ laser ablation, surgical excision, or modified shave technique [13,14,15,16,17,18,19,20,21,22,23,24,25]. Treatment planning should consider tumor number, size, location, cosmetic sensitivity of the area, scarring risk, and recurrence potential [26,27]. A new systemic treatment option—selumetinib, a MEK inhibitor—has been approved for NF1 by the FDA (2020) and EMA (2021), which is mainly used for symptomatic, inoperable plexiform neurofibromas in children [6,28]. Several new therapies are under evaluation, including systemic agents (everolimus, chloroquine, hydroxychloroquine), intralesional injections (1% deoxycholic acid, 1% polidocanol), mast cell–targeting treatments (tranilast, ketotifen), photodynamic therapy, and NFX-179 topical gel [29,30,31].

In recent years, novel, noninvasive imaging techniques, primarily used in dermatooncology, have revolutionized many fields of dermatology. These include high-frequency ultrasound (HFUS), optical coherence tomography (OCT), reflectance confocal microscopy (RCM), line-field confocal optical coherence tomography (LC-OCT), and multispectral imaging (MSI) [32,33,34,35,36]. The integration of artificial intelligence-based image analysis into these modalities holds promise for automated detection, classification, and longitudinal monitoring of neurofibromas, potentially improving diagnostic accuracy and enabling large-scale, standardized disease surveillance [37,38,39].

Dermoscopy-guided high-frequency ultrasound (DG-HFUS) is a newly developed portable device capable of providing simultaneous visualization of dermoscopic and HFUS images [40]. This feature facilitates precise positioning, thereby potentially enhancing the reproducibility of dermatologic examinations [41], a goal explicitly emphasized in Position Statement 5 of the European Federation of Societies for Ultrasound in Medicine and Biology (EFSUMB) on dermatologic ultrasound [42].

In this study, we aimed to explore the structural features of neurofibromas in NF1 patients using DG-HFUS and to identify objective parameters that could help guide diagnosis, monitor progression, and support treatment planning.

## 2. Materials and Methods

### 2.1. Inclusion and Exclusion Criteria

The inclusion criteria for this study involved obtaining informed consent from participants and confirming the diagnosis of NF1 based on the current criteria published by Legius et al. in 2021 [10], followed by molecular genetic analysis of the NF1 gene in all patients. Exclusion criteria were uncertain NF1 diagnosis (for example, Legius syndrome), lesions inaccessible for imaging due to body contour (skin folds, acral areas, genital region) or hair-covered areas, and lesions >10 mm due to equipment field-of-view limitations. Acral regions were excluded only in cases when they were practically inaccessible for imaging due to the body contour.

In patients presenting with a high number of cutaneous neurofibromas, a predefined number (5–10) of lesions per patient was selected for evaluation. These lesions were randomly selected from clinically eligible neurofibromas, aiming to represent different lesion sizes and anatomical locations. In patients presenting with fewer than 5 neurofibromas, all lesions were evaluated.

We assessed only localized cutaneous and subcutaneous neurofibromas. Plexiform, diffuse, and intraneural neurofibromas were not included in the present study.

### 2.2. DG-HFUS Imaging

From April 2023 to June 2024, measurements were performed at the Department of Dermatology, Venereology and Dermatooncology, Semmelweis University, using a portable DG-HFUS device (Dermus SkinScanner, Dermus Ltd., Budapest, Hungary). This system combines dermoscopic and HFUS imaging (33 MHz, range 20–40 MHz) to enhance diagnostic accuracy and reproducibility. The device features a silicone-membrane imaging window, gel coupling, and an optical module for precise lesion localization. Dermoscopic and ultrasound images are displayed side-by-side on a connected smartphone, with a 2-s acquisition time. Dermoscopy provides a 12 × 12 mm^2^ field of view (10× magnification), while ultrasound images extend 12 mm laterally and penetrate up to 10 mm. A red line marks the cross-sectional plane, and color coding aids structural visualization (the lowest intensities appear in dark shades, followed by green and blue, while the highest intensities are indicated by red and yellow) [41]. Recorded images were analyzed in SkinAid cloud software, stored under patient ID with demographic and lesion details. DG-HFUS assessments included size, location, shape, contours, surface, echogenicity, global echogenicity, and posterior acoustic features. At least five cross-sectional images were obtained per lesion.

## 3. Results

### 3.1. Patient and Lesion Characteristics

A total of 100 lesions, with a mean of 7.14 per patient, were evaluated from 14 patients with NF1. The cohort included 7 males and 7 females, with a mean age of 45 ± 15.87 years, and all had a diagnosis confirmed by molecular genetic analysis of the NF1 gene. Neurofibromas were typically located along peripheral nerves and were soft and compressible, features that help distinguish them from malignant lesions.

### 3.2. Clinical Presentation

The severity of symptoms among the examined patients (*n* = 14) showed considerable variability, which can be attributed to the variable expressivity of the disease. Café-au-lait macules (CALMs), axillary and inguinal freckling, as well as cutaneous and subcutaneous neurofibromas, were present in nearly all individuals; however, their number and size varied significantly between patients. Plexiform neurofibromas were observed in 43% of cases. Among ophthalmological findings, Lisch nodules were the most frequent, while scoliosis was the most common musculoskeletal manifestation. Malignancies occurred in three patients, specifically breast cancer, pheochromocytoma, and gastrointestinal stromal tumor (see Table 1).

The clinical characteristics according to the revised 2021 diagnostic criteria [10] are summarized for each patient in Table 2. The patients’ sex, age, age of onset of first symptoms, cutaneous and extracutaneous manifestations, and comorbidities are presented in Table 3.

### 3.3. DG-HFUS Imaging of Neurofibromas

**Figure 1:**
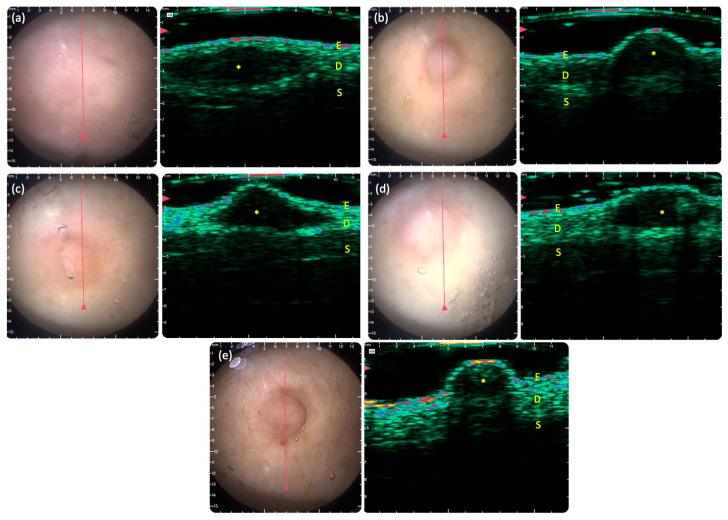
Dermoscopy-guided high-frequency ultrasound (DG-HFUS) images illustrating various shapes and posterior acoustic features of neurofibromas. (**a**) Ovoid shape; (**b**) round shape; (**c**) spiked shape; (**d**) posterior acoustic enhancement; (**e**) posterior acoustic shadowing. In the DG-HFUS images, the epidermis (E) appears as a thin, high-intensity band, and the dermis (D) shows lower echogenicity with occasional focal hyperintense areas. Neurofibromas (*) present as well-defined, hypoechoic structures located in the dermis or extending into the superficial subcutis. D: Dermis E: epidermis, S: subcutis.

DG-HFUS revealed that 79% of lesions were cutaneous neurofibromas (Figure 1b–e) and 21% were subcutaneous (Figure 1a). We classified a lesion as subcutaneous when more than 50% of the lesion was located in the subcutis. Based on clinical examination, shapes included ovoid (63%), round (28%), and spiked (9%) (Figure 1). The mean maximum vertical and lateral diameters were 5.37 ± 2.66 mm and 2.28 ± 1.39 mm, respectively. All lesions were hypoechoic; 62% had a homogeneous echotexture, while 38% were heterogeneous. Margins were well-defined in 57% and poorly defined in 43%. Surface elevation was present in 58% of lesions, whereas 42% appeared flat. Posterior acoustic enhancement was observed in 3% of cases, characterized by increased echogenicity distal to the lesion, and acoustic shadowing occurred in 10%, characterised by ultrasound signal attenuation beyond the lesion (Figure 1). The main results are summarized in Table 4.

## 4. Discussion

The morphological evaluation of neurofibromas in NF1 patients using DG-HFUS, as conducted in this study, may offer clinically relevant information that enhances diagnostic accuracy and supports therapeutic decision-making in everyday dermatologic practice.

The diagnosis of NF1 is primarily based on clinical findings [10]. However, these features often emerge progressively, and not all symptoms may be present at the time of examination. Although molecular genetic testing is increasingly used to confirm the diagnosis, it remains an expensive and time-consuming method, and not all pathogenic mutations have been identified to date. In cases where the patient presents with only a few neurofibromas, other lesions should be considered before establishing the diagnosis. As described above, neurofibromas typically exhibit a homogeneous and hypoechoic internal structure on ultrasound images, with well-defined but non-encapsulated margins. These sonographic features can assist in differentiating them from other subcutaneous or dermal lesions such as lipomas, epidermoid cysts, or dermatofibromas [43]. Furthermore, DG-HFUS allows for precise localization of the lesion within the dermis or subcutaneous tissue, which contributes to the differential diagnosis, as certain lesions show a predilection for specific skin layers.

HFUS imaging is also critical for identifying malignant peripheral nerve sheath tumors [44], which demand fundamentally different surgical management [45]. These malignancies most often develop from pre-existing plexiform neurofibromas, while cutaneous and subcutaneous neurofibromas are rarely associated with malignant transformation [46]. While magnetic resonance imaging (MRI) remains the gold standard for detecting and monitoring these lesions [46], DG-HFUS provides a valuable, non-invasive complementary tool, particularly well-suited for assessing superficial components. According to Rafailidis et al., HFUS reveals malignant peripheral nerve sheath tumors (MPNSTs) as fusiform, hypoechoic, and inhomogeneous lesions with continuity to a peripheral nerve [47]. These tumors frequently exhibit a partially thickened, irregularly hyperechoic peripheral rim, corresponding to a pseudocapsule. The borders may appear poorly defined in certain cases. Additional ultrasonographic features that should raise suspicion for malignancy include a lesion diameter exceeding 5 cm, poorly defined borders, central necrosis or hemorrhage, perilesional edema, and the presence of calcifications. Nonetheless, these findings are not pathognomonic and should be interpreted within the broader clinical context. Color, power, and spectral Doppler imaging could provide further information by visualizing abnormal vascular patterns, which indicate malignancy. These include vessel occlusion, stenosis, arteriovenous shunting, trifurcation, and tortuous vascular loops [47]. In malignant cases, the primary treatment is radical resection, aiming for complete tumor removal with wide safety margins. Adjuvant radiotherapy and chemotherapy may also be necessary in advanced or metastatic cases [45].

When neurofibromas cause significant quality-of-life impairment or result in marked aesthetic or functional deformities, surgical excision is warranted. Several excisional techniques are available, and DG-HFUS can aid in selecting the most appropriate approach. According to Chamseddin et al., the optimal method depends on lesion number and size: in patients with over 100 neurofibromas, CO_2_ laser ablation is recommended for lesions >5 mm, while electrodessication is preferred for lesions ≤5 mm. For those with fewer than 100 lesions, tumors >2 cm are best treated by in toto excision, whereas modified shave technique or photocoagulation is suitable for lesions <2 cm [26,27]. Experience from the Department of Dermatology, Venereology and Dermatooncology at Semmelweis University indicates that in toto excision brings the most favorable outcomes even for neurofibromas larger than 1 cm but smaller than 2 cm. Images obtained with the Dermus SkinScanner enable precise measurement of both lateral and vertical tumor diameters, which plays a critical role in determining the optimal excision method. DG-HFUS offers a comprehensive visualization of the lesion’s borders relative to adjacent tissues, thereby enabling more accurate delineation of tumor margins and minimizing unnecessary excision of uninvolved tissue [40]. In conclusion, the integration of HFUS imaging into the surgical management of neurofibromas contributes to improved aesthetic and functional outcomes.

In patients with NF1, neurofibromas typically manifest during puberty, with both their number and size potentially increasing progressively with age [7]. The application of DG-HFUS imaging offers a reliable modality for monitoring the progression of cutaneous lesions, as it enables precise documentation and reproducible measurements facilitated by optical guidance. While certain studies have reported an increase in the number and size of neurofibromas during pregnancy [7,48], other investigations have not identified statistically significant changes throughout gestation [49]. Consequently, DG-HFUS imaging could be valuable in clarifying this issue, as it allows detection and quantification of even the smallest changes during follow-up.

OCT is a non-invasive imaging modality that employs low-coherence interferometry with near-infrared light to acquire high-resolution cross-sectional images of cutaneous structures in real time. It provides excellent axial resolution (3–15 μm), enabling detailed visualization of the epidermis and superficial dermis. Despite its diagnostic utility, the technique is limited by a relatively shallow penetration depth (2 mm) and its high cost [34,35,50]. RCM utilizes a low-power laser to obtain high-resolution in vivo images of both the superficial and deeper layers of the skin [34,35]. Its principle is based on the interaction of monochromatic light with endogenous chromophores (e.g., melanin, hemoglobin), which reflect, scatter, or absorb the light depending on their optical characteristics. RCM achieves a lateral resolution of 0.5–1 μm and an axial resolution of 3–5 μm [34,35]. However, its penetration depth is relatively limited, typically ranging from 0.15 to 0.2 mm depending on the anatomical location [51]. MSI involves illuminating the skin with light beams of various wavelengths and capturing the reflected light using a highly sensitive detector. The technique primarily utilizes the visible and near-infrared regions of the electromagnetic spectrum (400–950 nm), with illumination provided by halogen lamps or LEDs. Since different skin components absorb and reflect light differently depending on the wavelength, the resulting spectral data enable detailed analysis of skin structure and composition [52]. Compared to the imaging modalities presented above, DG-HFUS offers numerous advantages. In contrast to OCT and RCM, DG-HFUS provides greater penetration depth, and it also outperforms these techniques in terms of portability, cost-effectiveness, and acquisition speed.

A major strength of our study is the application of a novel imaging modality that has not yet been widely adopted for the evaluation of cutaneous manifestations in NF1 patients. To the best of our knowledge, no previous studies have reported the use of dermoscopic guidance during ultrasonographic examination of neurofibromas.

The limitations of our study include the relatively small sample size, which is attributable to the rare nature of the disease. Another limitation of DG-HFUS is that its clinical applicability is highly dependent on operator expertise, and the measurements may be subjective to a certain extent. Additionally, the technique is not applicable in certain anatomical locations, and its field of view restricts the maximum area that can be imaged. Lesions larger than 10 mm need to be assessed by other imaging techniques in future studies. The examples shown in the images of Figure 1 present hypoechoic structures below 6–7 mm. In our experience, the DG-HFUS device utilized in this study is capable of visualizing deeper structures (e.g., echoes from bones) from up to 10 mm depth, whereas for many body locations, the tissues beneath the dermis appear as strongly hypoechoic, such as those in the images presented here. In addition, it is possible to change the brightness of the ultrasound images of the device within the application. Higher brightness settings usually help visualize signals from below 7 mm as well. However, in this study, lower brightness settings were intentionally applied to optimize image contrast and enable more accurate morphological assessment of neurofibromas.

## 5. Conclusions

NF1 is a multisystem genodermatosis with variable expressivity [53] and reduced life expectancy, primarily due to increased tumor risk [54]. Tumor surveillance is therefore essential, while neurofibromas also significantly impair quality of life through aesthetic, sensory, and functional symptoms [8]. Our findings show that DG-HFUS is a valuable, non-invasive tool for assessing cutaneous and subcutaneous neurofibromas, providing eight objective imaging parameters, size, location, shape, contours, surface, echogenicity, global echogenicity, and posterior acoustic features, that support diagnosis, surgical planning, and follow-up. By defining lesion dimensions and tissue relationships, DG-HFUS aids in selecting optimal surgical techniques and monitoring morphological changes over time.

## Figures and Tables

**Table 1:** The prevalence of clinical manifestations among NF1 patients included in the study.

Symptoms	The Number and Percentage of Affected Patients
CALM	11 (79%)
Axillary and inguinal freckling	12 (86%)
Cutaneous and subcutaneous neurofibroma	14 (100%)
Plexiform neurofibroma	6 (43%)
Lisch-nodule	11 (79%)
Scoliosis	5 (36%)
Malignancy	3 (21%)

CALM: café-au-lait macule.

**Table 2:** Clinical data according to the revised 2021 diagnostic criteria.

Patients	CALM	Axillary or Inguinal Freckling	NF	Optic Glioma	Lisch Nodule	Skeletal Abnormality	NF1 Variant	Family History	Number of NF
NFP1	>6	✓	>2	-	>2	-	pos	neg	4
NFP2	>6	✓	>2	-	>2	-	pos	pos	4
NFP3	-	-	>2	-	-	-	pos	neg	7
NFP4	-	-	>2	-	-	-	pos	neg	7
NFP5	>6	✓	>2	-	>2	-	pos	neg	8
NFP6	>6	✓	>2	-	>2	-	pos	neg	9
NFP7	>6	✓	>2	-	>2	-	pos	pos	10
NFP8	>6	✓	>2	-	>2	-	pos	neg	6
NFP9	>6	✓	>2	-	-	-	pos	pos	5
NFP10	>6	✓	>2	-	>2	-	pos	neg	8
NFP11	>6	✓	>2	-	>2	-	pos	neg	7
NFP12	>6	✓	>2	-	>2	-	pos	pos	13
NFP13	>6	✓	>2	-	>2	-	pos	pos	3
NFP14	-	✓	>2	-	>2	-	pos	pos	9

CALM: café-au-lait macule; neg: negative; NF: neurofibroma; NF1: neurofibromatosis type 1; NFP: patient affected by neurofibromatosis; pos: positive.

**Table 3:** Epidemiological and clinical characteristics of the patients included in the study.

Patients	Sex Assigned at Birth	Age (Year)	Onset of Symptoms	Cutaneous Symptoms	Extracutaneous Symptoms	Comorbidites
NFP1	male	50	childhood	CALM, freckling, neurofiroma	Lisch nodules, hypertension	asthma bronchiale
NFP2	male	70	childhood	CALM, freckling, neurofiroma	Lisch nodules, pheochromocytoma, hypertension	asthma bronchiale, BPH, nephrolithiasis, gastritis chronica
NFP3	female	58	adulthood	neurofibroma	hypetension	struma nodosa
NFP4	female	52	adulthood	neurofibroma, nevus anemicus	Breast cancer, adrenal adenoma	-
NFP5	female	34	adolescence	CALM, freckling, neurofiroma	Lisch-nodules	migraine, myopia
NFP6	female	38	adolescence	CALM, freckling, neurofiroma	Lisch-nodules	cholelithiasis, gastric ulcer
NFP7	male	21	childhood	CALM, freckling, neurofiroma	Lisch nodules, scoliosis	-
NFP8	male	19	childhood	CALM, freckling, neurofibroma, juvenile xanthogranuloma	Lisch nodules, scoliosis	-
NFP9	female	35	childhood	CALM, freckling, neurofibroma	scoliosis	cataracta, myopia, allergic rhinitis
NFP10	male	31	childhood	CALM, freckling, neurofibroma	Lisch nodules	myopia, strabismus
NFP11	male	73	adolescence	CALM, freckling, neurofibroma	Lisch nodules, scoliosis, hypertension, GIST	contact dermatitis
NFP12	female	56	adolescence	CALM, freckling, neurofibroma	Lisch-nodules	hyperlipidemia
NFP13	female	47	childhood	CALM, freckling, neurofibroma	Lisch nodules, scoliosis	spina bifida
NFP14	female	39	childhood	freckling, neurofibroma, nevus anemicus	Lisch nodules	-

CALM: café-au-lait macule; BPH: benign prostatic hyperplasia; GIST: gastrointestinal stromal tumor; NFP: patient affected by neurofibromatosis.

**Table 4:** Ultrasound characteristics of 100 neurofibromas assessed with a dermoscopy-guided 33 MHz high-frequency ultrasound device.

**Location**	Dermis	79/100 (79%)
	Subcutis	21/100 (21%)
**Shape**	Round	28/100 (28%)
	Ovoid	63/100 (63%)
	Spiked	9/100 (9%)
**Global echogenicity**	Hypoechoic	100/100 (100%)
	Hyperechoic	0/100 (0%)
**Echogenicity**	Homogenous	62/100 (62%)
	Heterogenous	38/100 (38%)
**Contour**	Well-defined	57/100 (57%)
	Poorly defined	43/100 (43%)
**Posterior acoustic feature**	Enhancement	3/100 (3%)
	Shadowing	10/100 (10%)
**Surface**	Protruding	58/100 (58%)
	Flat	42/100 (42%)

## Data Availability

The data presented in this study are available upon request from the corresponding author.

## Abbreviations

CALM	café-au-lait macules
CNV	copy number variation
DG-HFUS	dermoscopy-guided high-frequency ultrasound
EFSUMB	European Federation of Societies for Ultrasound in Medicine and Biology
HFUS	high-frequency ultrasound
LC-OCT	line-field confocal optical coherence tomography
MPNST	malignant peripheral nerve sheath tumors
MRI	magnetic resonance imaging
MSI	multispectral imaging
NF1	neurofibromatosis type 1
OCT	optical coherence tomography
QOL	quality of life
RCM	reflectance confocal microscopy
